# Effect of EndoActivator and Er,Cr:YSGG laser activation of Qmix, as final endodontic irrigant, on sealer penetration: A Confocal microscopic study

**DOI:** 10.4317/jced.53270

**Published:** 2017-02-01

**Authors:** Sarika Chaudhry, Sudha Yadav, Sangeeta Talwar, Mahesh Verma

**Affiliations:** 1 MDS, Associate Professor, Department of Conservative dentistry & Endodontics, Maulana Azad Institute of Dental Sciences, New Delhi- 110002, India; 2 MDS, Senior resident, Department of conservative dentistry and endodontics, maulana azad institute of dental sciences, New Delhi- 110002, India; 3 MDS, Professor & Head of Department, Department of Conservative dentistry & Endodontics, Maulana Azad Institute of Dental Sciences, New Delhi- 110002, India; 4 MDS, Director-Principal, Department of Conservative dentistry & Endodontics, Maulana Azad Institute of Dental Sciences, New Delhi- 110002, India

## Abstract

**Background:**

Through chemomechanical debridement of the root canal is a primary requisite for successful endodontic therapy. Thus the aim of this study was to evaluate the effects of using QmiX alone, QmiX with EndoActivator and QmiX with Er,Cr:YSGG laser for final irrigation on sealer penetration into the dentinal tubules.

**Material and Methods:**

75 extracted human mandibular premolar teeth were treated with sodium hypochlorite (NaOCl) irrigation. The samples were divided into 5 groups according to the final irrigation solution used: (1) 17% EDTA and 2.5% NaOCl, (2) QmiX (3) QmiX with Er,Cr:YSGG laser and (4) QmiX with EndoActivator (5) 2.5%NaOCl. All teeth were obturated using cold lateral condensation technique with gutta percha and AH 26 sealer (Dentsply; DeTrey,Konstanz, Germany) labeled with Rhodamine B dye. The teeth were sectioned at distances of 2 and 5 from root apex. Total percentage and maximum depth of sealer penetration were measured using confocal laser scanning microscopy.

**Results:**

Results of one way Anova analysis showed that there was a significant difference in the percentage and depth of sealer penetration among all groups at 3 and 5 mm level sections (*P* < .05). Within the groups maximum sealer penetration was recorded for Er,Cr:YSGG laser activated group. Greater depth of sealer penetration was recorded at 5mm as compared to 3mm in all the groups.

**Conclusions:**

Activation of QMix using EndoActivator and Er,Cr:YSGG laser enhanced the sealer penetration at apical and middle third. Thus Er,Cr:YSGG laser and EndoActivator may act as an appropriate adjunct during chemomechanical preparation of the root canal.

** Key words:**EndoActivator, Er,Cr:YSGG laser, Qmix, confocal microscopy, sealer penetration.

## Introduction

An ideal endodontic irrigant should have the ability to dissolve organic and inorganic matter of the root canal, possess antibacterial properties and should be safe for the surrounding tissue (1). Sodium hypochlorite (NaOCl) and 17% EDTA in conjugation are the most commonly employed irrigants. NaOCl mainly acts on the organic content whereas EDTA dissolves the inorganic portion of dentin. However it was reported that when NaOCl is used after EDTA as final irrigant, it results in marked erosion of dentine (2). Prior ethical approval was taken from the ethic committe. Because of the recognized limitations of available irrigants, tremendous research is being done to develop an ideal final irrigant which meet all standards.

QMiX (Dentsply Tulsa Dental, Tulsa, OK) is a novel final endodontic irrigant. It is a solution containing EDTA, Chlorhexidine (CHX) and a detergent. In addition to its good antimicrobial properties it also removes smear layer efficiently. No precipitate formation is seen in QmiX when EDTA and CHX are mixed together because of its unique chemical design. Also despite the CHX content, mixing QMiX with NaOCl does not produce any precipitate (3).

Several devices have been introduced to increase the efficacy of irrigation, including sonic/ultrasonic devices and different types of lasers. Recently, the EndoActivator System (Dentsply Tulsa Dental Specialties, Tulsa, OK) which is a sonically driven irrigation system was introduced. It comprises of disposable flexible polymer tips of 3 different sizes that do not cut root dentin. By the means of vigorous intracanal fluid agitation, it effectively activates the irrrigant (4).

Lasers are being used for nonsurgical endodontic treatment since the early 1970s still its acceptance has been slow due to lack of knowledge and proper education. Laser treatment can be a valuable tool for the removal of the dentinal smear layer as it activates the irrigation solution by the transfer of pulsed energy. Erbium, chromium: yttrium, scandium, gallium garnet (Er,Cr:YSGG) laser (Waterlase MD, Biolase, San Clemente, USA) has a 2,790 nm wavelength delivered by using radial firing tips. This wavelength has highest absorption in water and high affinity to hydroxyapatite (5).

If smear layer has been removed efficiently, sealer often penetrates into dentinal tubules thus attacking the residual microbes and provides a fluid tight seal (6). To study the distribution of sealer inside dentinal tubules, confocal scanning microscope (CLSM) is an excellent tool. It has the advantage of providing detailed information at low magnifications (10X) through the use of fluoroescent rhodamine marked sealers (7).

Actually, this is the first investigation which was done to evaluate the influence on depth of sealer penetration with latest endodontic irrigation device, i.e., EndoActivator system and hard tissue laser, i.e., Er,Cr:YSGG laser, with newly available Qmix as an effective endodontic irrigant using confocal scanning microscopy. The null hypothesis tested was that the use of EndoActivator system and Er,Cr:YSGG laser did not improve the depth of sealer penetration into dentinal tubules when Q Mix was used as final irrigant.

## Material and Methods

-Preparation of the Teeth

75 single rooted human mandibular premolar teeth were used in the study. All teeth were stored in physiological saline solution until use. Teeth were decoronated at cementoenamel junction with a high speed fissure bur under water cooling. A #10 K-file was introduced into the canal and advanced until it was visible at the apex. It was retracted 1 mm to establish the working length. After coronal preflaring, the apical half of the canal was prepared using stepback technique up to a master file size 40. The root canals were irrigated with 2.5% sodium hypochlorite solution using a 27 gauge Endodontic Needle (Monoject, Sherwood Medical, St Louis, MO, USA) during instrumentation.

After chemomechanical preparation, the teeth were randomly divided into 5 groups of 15 teeth each according to the final irrigation protocol.

(Group 1) Final rinse with 5 ml 17% EDTA (Pulpdent EDTA Solution 17%, Pulpdent Corporation, Watertown, MA, USA) for 3 minutes followed by 2.5% NaOCl.

(Group 2) Final rinse with 5 mL QMix for 1 minute.

(Group 3) Final rinse of 5 mL QMix. Er,Cr:YSGG (Millennium Biolase Technology Inc., San Clemente, CA, USA) was used for activation of QMix. Samples were irradiated with an Er,Cr:YSGG laser at a wavelength of 2.796 µm with a flexible fiber [diameter 300 µm - Z3 Endolase (Biolase)]. The operating parameters used were: 1.25 W, 20Hz, The flexible fiber was inserted into the root canal 1mm short of the working length. During irradiation, the fiber tip was moved in a spiral motion along the root canal walls. The procedure was repeated five times for 5 s with a time interval of 20 s (8).

(Group 4) Final rinse of 5 mL QMix. EndoActivator was used to activate the irrigant for 60 s at 10,000 cycles per min with a size 25/.04 polymer tip.

(Group 5) Final rinse with 2.5% sodium hypochlorite.

Group 1-4 acted as experimental groups while group 5 was taken as control

-Root Canal obturation

 All root canals were dried with paper points. A standardized #40 size gutta percha cone (Dentsply Maillefer) with tug back was selected. Root canal sealer AH 26 (Dentsply Detrey, Konstanz, Germany) was mixed according to the manufacturer’s instructions. It was mixed with Rhodamine B dye to an approximate concentration of 0.1% in order to allow visualization under confocal microscope. It was placed into the canal till the working length with the gutta percha. The master cone was then coated with root canal sealer and gently placed at the working length. Lateral condensation was carried out using size 20 and 25 accessory gutta-percha cones with endodontic finger spreaders (Dentsply Maillefer) placed within 2 mm of the working length. Lateral condensation was continued until accessory cones could not be introduced more than 3mm into the root canal. Following obturation, the gutta-percha was removed to the level of the cementoenamel junction with a warm instrument.

-Confocal Laser Scanning Microscopic analysis

**Figure 1:**
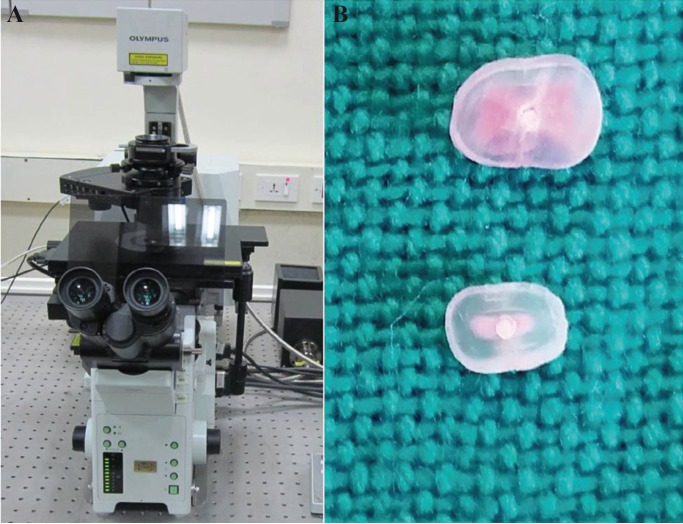
Confocal microscope A) and transverse sections B) at 3 and 5 mm level.

Each root was sectioned horizontally at distances of 3 and 5 mm from the apical tip with a diamond saw rotating at 500 rpm under constant water cooling (Mecatome T201 A; Presi, Tavernoles, France). Specimen surfaces were polished using sandpaper. The sections were examined under an Olympus FV1000 confocal laser scanning microscope (Olympus FluoView™ FV1000). Figure 1(A-B) shows the confocal microscope and the tooth sections (at 3 and 5mm level) respectively. Images obtained from confocal microscopy were analyzed using Adobe Photoshop 7.0 (Adobe Systems, Inc, San Jose, CA). The circumference of the root canal wall was outlined and measured with the measuring tool available in software. Then areas along the canal walls which showed sealer penetration into dentinal tubules were measured. The measured distances were divided by the total canal circumference to calculate the percentage of the area of canal wall covered by sealer. For measuring the depth of penetration, the point of deepest penetration was measured from the canal wall to the point of maximum sealer penetration.

-Statistical Analysis

Statistical analysis was performed using one way Anova for comparisons between groups. Post hoc Tukey test was used for comparisons within groups. The level of significance was set at *P* < .05.

## Results

**Figure 2:**
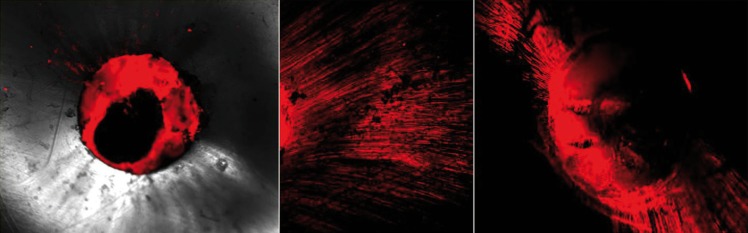
Representative confocal laser scanning microscopic images at 3 mm level for control group A) showing no sealer penetration; B) Er,Cr:YSGG laser activated group showing extensive sealer penetration; C) Endoactivator group.

Mean percentage and depth of sealer penetration at 3 and 5mm level in shown in table  Table 1 and  Table 2 respectively. One way Anova analysis showed that there was a significant difference in the percentage of sealer penetration among all final irrigation groups at 3 and 5mm level (*P* < .05). However no significant difference was seen in the percentage of sealer penetration between all experimental groups in the sections of both the levels. The control group showed a significantly lower percentage of sealer penetration than the other groups in all sections (*P* < .05). Highest depth of sealer penetration was seen in group 4( Er,Cr:YSGG activated QMix) followed by group 3 (EndoActivator). Greater depth of sealer penetration was present in the 5mm section as compared to 3mm irrespective of the irrigation protocol. There were significant differences between the apical and middle sections in all experimental groups but not in the control group. Confocal images showing sealer penetration in the dentinal tubules are depicted in figure 2.

**Table 1:**
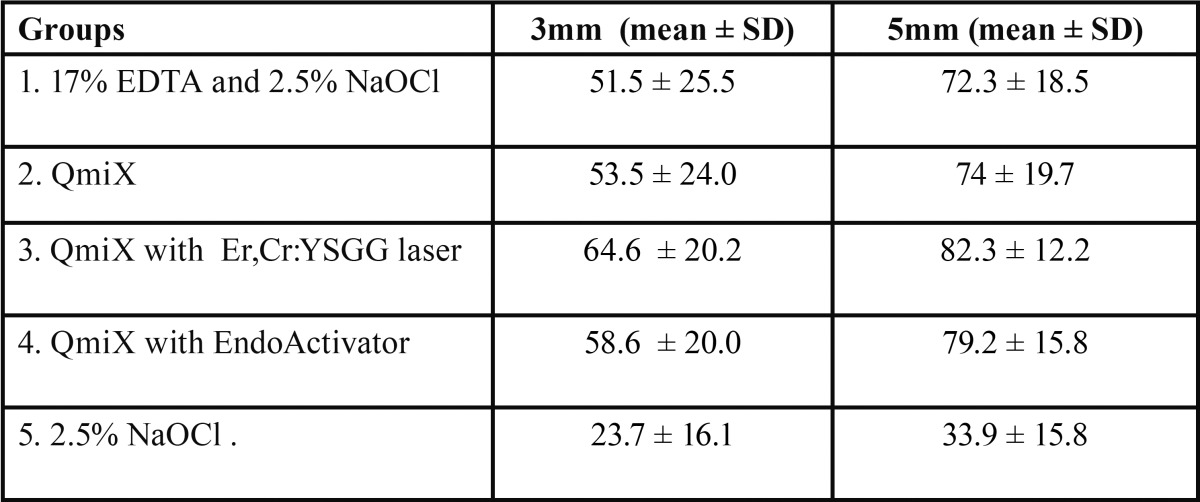
The Percentage of Sealer Penetration (%) into Dentinal Tubules at the 3 and 5mm sections.

**Table 2:**
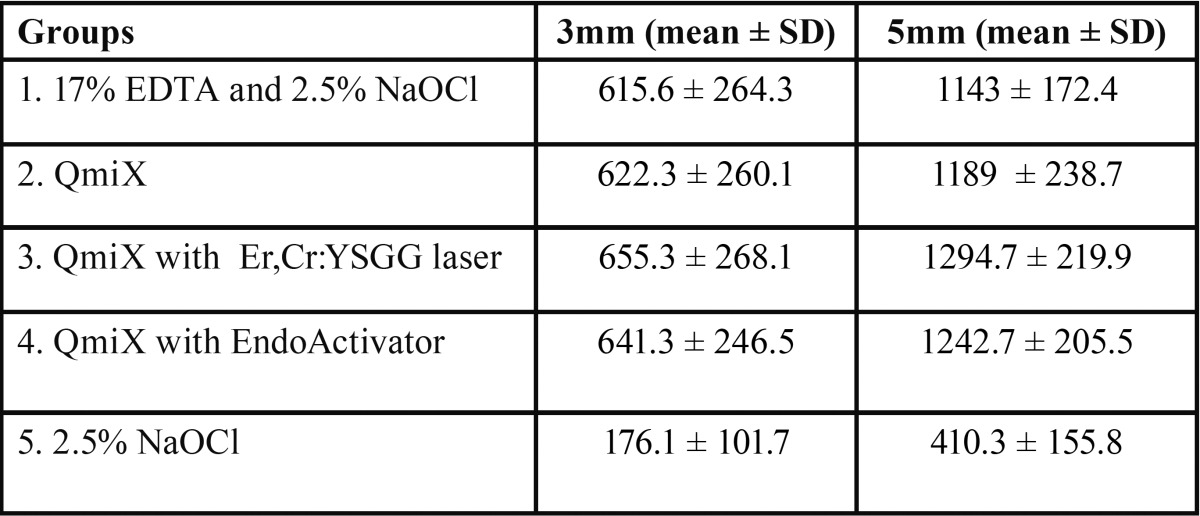
The Maximum Depth of Sealer Penetration (in µm) into Dentinal Tubules at 3 and 5mm level.

## Discussion

For successful endodontic treatment, complete debridement and decontamination of the root canal system is an absolute necessity. Mechanical instrumentation and irrigation play crucial role in achieving this goal (9). However in apical third it is very difficult to completely eradicate microbes because of presence of isthmuses, anastomoses and oval extensions (10). Hand irrigation alone is often insufficient in removing debris and smear layer from the apical third (10). Therefore activation of the irrigants is necessary to increase the efficacy of irrigant. The removal of smear layer influences the sealer penetration and enhances the interface between obturation material and canal walls (11).

Disinfecting solutions are often used after removal of smear layer for eradicating deep seated microbes in the dentinal tubules. QMix when used as a final irrigant removes smear layer and exerts antimicrobial action simultaneously because of presence of CHX and EDTA in its composition. CHX gets adsorbs onto dentin surface and prevents microbial colonization. A recent study showed QmiX to be as effective as 6% sodium hypochlorite against *E. faecalis* in dentinal tubules (12). Because of the excellent properties exhibited by QMix in all these researches, QMix was taken as final irrigant in present study.

EndoActivator, a sonically driven irrigation system, has been shown to remove smear layer and reduce bacterial load (13). EndoActivator, through acoustic streaming and cavitation, produces agitation of the irrigants. This hydrodynamic activation improves penetration of irrigant into the root canal system (14). In 2002, Er,Cr:YSGG laser was approved by the US Food and Drug Administration (FDA) for use in endodontic therapy. Despite of the availability of lasers in dentistry since many years, very little research has been carried out to assess its efficacy in the field of endodontics (15). In literature there are not many studies that have compared the depth of sealer penetration after activation of irrigant using EndoActivator and Er,Cr:YSGG laser. In our study Er,Cr:YSGG laser activated group showed greatest sealer penetration at both 3 and 5 mm level. Also the use of EndoActivator resulted in better sealer penetration indicating its ability to penetrate and clean the inaccessible areas of root canal system. Thus null hypothesis was rejected, as the depth of sealer penetration into the dentinal tubules was effected by EndoActivator and Er,Cr:YSGG activation of QMix.

In literature there are conflicting views regarding the use of lasers (16,17). Certain adverse effects like thermal injury including carbonization and partial melting with the use of laser has been reported in literature. It might be due to variation in power settings without using water spray cooling (18). That is why before the use of any kind of laser it is very important to understand the mechanism and have proper knowledge regarding the parameters needed for the use of the particular laser. The obturation of a greater number of root canal ramifications using gutta-percha and/or sealer after treatment with Er,Cr:YSGG following mechanical instrumentation has also been demonstrated (19). The activation of irrigant with Er,Cr:YSGG laser is also found to be effective method in smear layer removal (20). A “shockwave-like” effect is observed when spiral tips of laser are submerged in a liquid filled root canal. This effect may remove the smear layer and clogged debris from the canal and improve sealer penetration.

Attempts were made to minimize operator bias in this study. Root canal walls were coated with sealer using gutta percha points by a single, skilled operator (21). Image analysis was also carried by a single operator. Cold lateral compaction was used in this study because of its simplicity and universal acceptance. It is a practical and reliable technique for root canal obturation and doesn’t require any sophisticated armamentarium (22).

The three-dimensional seal depends upon how extensively sealers penetrates into the dentinal tubules (21). Several previous studies (23,24) have shown that coronal third has greatest sealer penetration. The results of our study also indicate the same as the depth of penetration of sealers was more at the 5mm section as compared to the apical third irrespective of the irrigation protocol. This might be because of better accessibility of middle third to irrigants resulting in efficient removal of smear layer and debris. Also dentinal tubules are wider and more in number in coronal/middle third as compared to apical third (25). For this reason, the use of a laser may be more beneficial in the apical third to facilitate the smear layer removal in this area. Thus, under the tested conditions, EndoActivator and Er,Cr:YSGG laser enhances the sealer penetration into the dentine tubules specially in the apical third, which is the most inaccessible area to instrumentation.

## Conclusions

Under the parameters of this study, the use of the EndoActivator and Er,Cr:YSGG laser activated QMix significantly improved the sealer penetration at apical and middle third. Er,Cr:YSGG laser and EndoActivator demonstrated its potential to act as safe and faster option for debriding the root canal system in a minimally invasive manner. However, more research is needed before recommending its widespread use.
